# Bi-Component Nanostructured Arrays of Co Dots Embedded in Ni_80_Fe_20_ Antidot Matrix: Synthesis by Self-Assembling of Polystyrene Nanospheres and Magnetic Properties

**DOI:** 10.3390/nano7090232

**Published:** 2017-08-23

**Authors:** Marco Coïsson, Federica Celegato, Gabriele Barrera, Gianluca Conta, Alessandro Magni, Paola Tiberto

**Affiliations:** 1 INRIM, Nanoscience and Materials Division, Strada delle Cacce 91, 10135 Torino, Italy; f.celegato@inrim.it (F.C.); g.barrera@inrim.it (G.B.); gianluca.conta@gmail.com (G.C.); a.magni@inrim.it (A.M.); p.tiberto@inrim.it (P.T.); 2 Chemistry Department, Università di Torino, via Pietro Giuria 7, 10125 Torino, Italy

**Keywords:** bi-component nanostructured system, self-assembling, magnetic coupling

## Abstract

A bi-component nanostructured system composed by a Co dot array embedded in a Ni_80_Fe_20_ antidot matrix has been prepared by means of the self-assembling polystyrene nanospheres lithography technique. Reference samples constituted by the sole Co dots or Ni_80_Fe_20_ antidots have also been prepared, in order to compare their properties with those of the bi-component material. The coupling between the two ferromagnetic elements has been studied by means of magnetic and magneto-transport measurements. The Ni_80_Fe_20_ matrix turned out to affect the vortex nucleation field of the Co dots, which in turn modifies the magneto-resistance behaviour of the system and its spinwave properties.

## 1. Introduction

Composite magnetic materials have been the subject of intensive research both for fundamental investigations and applied research. In recent decades, the interplay among magnetic phases having different coercivity values and their exchange coupling (i.e., soft/hard magnetic nanocomposites, nanocrystalline soft grains dispersed in an amorphous magnetic matrix) has been deeply investigated. These investigations have led to viable soft magnetic alloys for magnetic transformers and soft/hard magnetic systems for very competitive permanent magnets [1,2].

Generally, nanocomposites are constituted by coupling different materials, and at least one displays magnetic properties. They can come in the form of magnetic particles dispersed in non-magnetic media (either metallic or insulating) [3,4], core–shell nanoparticles where the magnetic core is coated with a layer of antiferromagnetic or ferro/ferrimagnetic shell [5,6], multilayers, and stacked or adjacent elements (superlattices) [7,8,9,10,11], magnonic crystals [12,13]. Depending on the chosen composition and shape (i.e., layer or nanoparticle), exchange coupling among different magnetic phases occurs and gives rise to different effects. Among these emerged long-range magnetostatic interactions [14] are exchange bias [15,16], or interface interactions [17,18,19]. In turn, these give rise to uncommon and artificial magnetic and magneto-transport properties [7,11,17,20,21,22], such as giant [19] and tunnel magneto-resistance [18], and spin oscillations affecting spin waves [10,22,23,24]. The variety of possible applications and needs in spintronics [25] requires the availability of different preparation techniques of such materials, each optimized for the most critical aspects, e.g., feature size, interface quality between the materials, low cost, high yield, etc. This quest has been partly achieved by the advent of nanolithographic techniques that opened up the design of a variety of magnetic nanostructures with functional properties [26]. In particular, the possibility to tune lattice geometry of patterning (i.e., honeycomb, rhomboid) gave rise to magnetoresistance response strongly dependent on lattice geometry [27]. In this context, self-assembling lithography techniques [28,29,30,31] have proven their viability for obtaining low-cost, large-area nanopatterned magnetic systems with interesting magnetic and magneto-transport properties.

In this paper, we exploit a self-assembling polystyrene nanospheres lithography process [31,32] to obtain a bi-component nanostructured system composed of Co dots dispersed in a Ni_80_Fe_20_ antidot matrix. Through magnetic and magneto-transport investigations, their mutual magnetic interactions will be discussed. Depending on the applications, the preparation technique and investigation methods discussed in this paper can be exploited to pick suitable magnetic materials to be coupled, and to optimise the dots size and their centre-to-centre distance in order to reach the desired effect.

## 2. Experimental

**Figure 1:**
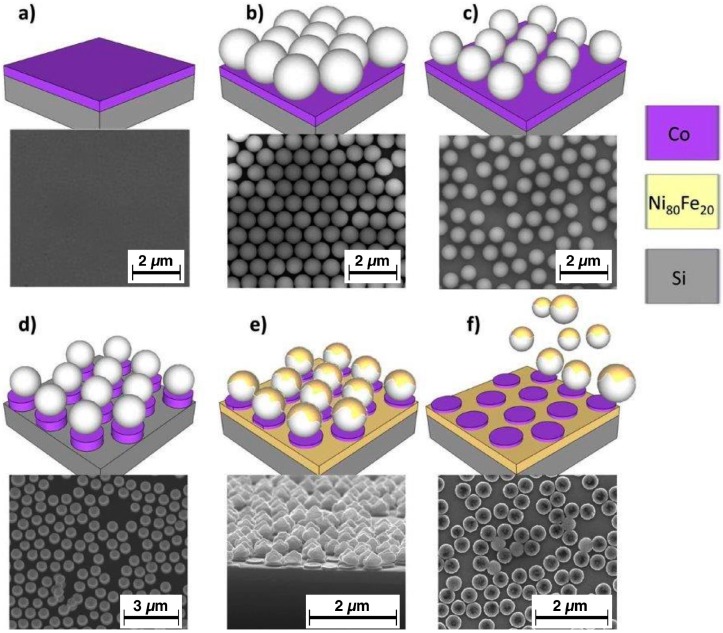
(Colour online) Scheme of the preparation process of Co dots and Ni_80_Fe_20_ antidot arrays constituting bi-component nanostructured systems, by polystyrene nanospheres lithography. (**a**) Initial continuous Co layer; (**b**) Polystyrene nanospheres self assembly; (**c**) Nanospheres diameter reduction; (**d**) Excess Co removal by Ar^+^ etching; (**e**) Ni_80_Fe_20_ deposition; (**f**) Nanospheres removal, final bicomponent structure.

Two-dimensional bi-component nanostructured (BN) arrays, constituted by Co dots embedded in a Ni_80_Fe_20_ antidot matrix (Co/Ni_80_Fe_20_), are synthesized by the self-assembling of polystyrene nanospheres (PN). This technique offers a low-cost, fast approach to obtain a large area patterned systems in forms of dots or antidots arrays, at the expense of a reduced degree of order of the patterns with respect to conventional lithography techniques (e.g., optical, electron beam), which seldom significantly affects their magnetic properties [33]. The multi-step fabrication process is schematically illustrated in Figure 1, and accompanied by the relevant scanning electron microscopy (SEM, FEI Inspect-F; the samples have been imaged as-is in high-vacuum using secondary electrons, without further preparation) images. Initially, a continuous Co magnetic thin film (thickness t_Co_ = 40 nm) is deposited by rf sputtering on a Si substrate covered with a native oxide layer (Figure 1a). Then, a monolayer of commercially available PN (starting diameter 500 nm) is deposited with the floating technique onto the Co thin film, resulting in a close-packed hexagonal lattice of nanospheres (Figure 1b). In the third step, the PN diameter is reduced by plasma etching in Ar^+^, by putting the sample in a floating potential into an ionized Ar^+^ plasma obtained in a vacuum chamber with a base pressure of ~10^−3^ mbar: the PN remain approximately in the same place, but their size is reduced as a function of the etching time; in the present case, their final diameter is ~350 nm. Subsequently, the polystyrene nanospheres are used as a hard mask for sputter etching with Ar^+^ ions the magnetic material (Co) that remains exposed among the nanospheres (Figure 1d). This time, the sample is placed in a vacuum chamber with a base pressure of ~10^−7^ mbar, and is used as an electrode (cathode) for the formation of the plasma, and the Ar^+^ ions remove the exposed magnetic material by bombardment. With a suitable calibration of the etching time, the Co layer can be completely removed, exposing again the substrate, and leaving an array of Co dots underneath the PN. Of these, the dots preserve the diameter (350 nm, determined during the size reduction process) and the centre-to-centre distance (500 nm, determined by the initial nanospheres diameter), whereas their thickness is equal to 40 nm as the continuous Co layer from which they have been obtained. The PN are then exploited a second time as a hard mask in the following step: a Ni_80_Fe_20_ layer (thickness t_NiFe_ = 30 nm) is deposited by rf sputtering on top of the PN, and among them on the substrate (Figure 1e). Finally, the PN are removed by sonication in deionized water (Figure 1f); the Ni_80_Fe_20_ deposit on top of them comes away with the nanospheres, whereas the material that has been deposited on the substrate among the nanospheres forms an antidot array. Therefore, the final sample is constituted by an Ni_80_Fe_20_ matrix in which circular holes arranged in a partially disordered hexagonal configuration are filled with slightly thicker Co dots. In the corresponding SEM image, some defects (e.g., vacancies, dislocations) are visible in the ideal hexagonal lattice of the final BN array, as is commonly the case when self-assembly techniques are used [30]. 

In addition to the BN array, two reference samples have also been prepared, one with an array of Co dots having similar diameter, centre-to-centre distance, and thickness, and one with a Ni_80_Fe_20_ antidot (AD) array, with comparable hole geometry and metallic layer thickness. Both have been produced by using the same self-assembling polystyrene nanospheres technique [32], and will serve as reference samples. Given the characteristics of the fabrication process, the Co dot and Ni_80_Fe_20_ antidot arrays of the reference samples shall be considered statistically equivalent to the respective components of the BN sample.

Hysteresis loops and first order reversal curves (FORCs) have been measured at room temperature on both the BN array and the reference samples by means of an alternating gradient field magnetometer (LakeShore 2900 AGFM, Princeton Measurement Corporation, Princeton, NJ, USA) operating in the field range −18 kOe < H < 18 kOe, with the magnetic field applied in the samples’ plane. Additionally, isothermal hysteresis loops have been measured in the temperature interval 5–300 K by a SQUID magnetometer (Quantum Design MPMS3 VSM-SQUID, Quantum Design, San Diego, CA, USA) operating in the field range of −70 kOe < H < 70 kOe, with the magnetic field applied in the samples’ plane. On cooling from room temperature down to 5 K, the samples have been either demagnetised (zero field cooling, ZFC) or submitted to a field of 10 kOe (field cooling, FC), before measuring the first hysteresis loop at 5 K. Subsequent loops have been measured at increasing temperatures up to 300 K.

Atomic force (AFM) and magnetic force microscopy (MFM) has been performed with a Bruker Multimode V Nanoscope 8 microscope (Bruker, Billerica, MA, USA) operated in intermittent contact/lift mode to image magnetisation configurations using a commercial ferromagnetic Co-Cr coated tip (MESP-HR, coercive field ≈900 Oe, Bruker, Billerica, MA, USA). Images of all samples have been acquired at the remanence after application of an in-plane saturating magnetic field. Images have been taken with a pixel resolution of ~10 nm with the oscillation frequency and amplitude set point obtained during tuning of the cantilever.

Magnetoresistance (MR) measurements at room temperature have been performed by means of a standard four-contacts technique at constant current intensity, both in the longitudinal (current parallel to magnetic field) and transverse (current perpendicular to magnetic field) configuration, under a maximum applied field H_max_ = 1 kOe.

**Figure 2:**
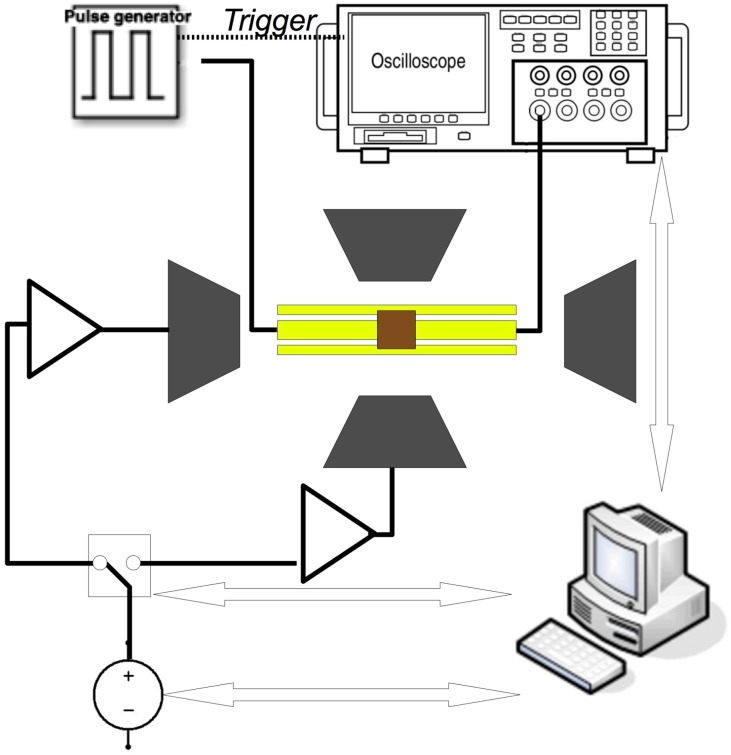
(Colour online) Scheme of the high-frequency, coplanar, waveguide-based measurement system.

The high-frequency measurements were performed by placing the samples face down on a coplanar waveguide (CPW) in an experimental setup that is schematically shown in Figure 2. The CPW has a ground-signal-ground structure with a signal line width of 50 μm, and a signal-ground gap of 20 μm. The CPW has been insulated by spinning an AZ5214E (Merck Performance Materials, Wiesbaden, Germany) resist layer approximately 1 μm thick to obtain the electrical insulation from the sample. The CPW is used to deliver short rise-time magnetic field pulses perpendicular to the waveguide, by a Picosecond 4050B pulse generator (10 V pulses with 45 ps rise time, 1 MHz repetition rate, Tektronix, Bracknell, UK). The sample, placed on top of the waveguide, is in the gap of a four-pole electromagnet for generating magnetic fields in arbitrary directions in the sample plane. For the measurement, the sample was subject at first to a background measurement under saturating transverse DC field H_ref_ = 62.5 Oe, parallel to H_pulse_, in order to provide a reference condition. The measurement is then repeated by applying a longitudinal bias field H in the range of 0–900 Oe. Each data acquisition is obtained by averaging 20 thousand measurements with a LeCroy Wavemaster SDA816Zi oscilloscope (LeCroy, Chestnut Ridge, NY, USA) acquired in sampling mode, at a data point resolution of 5 ps. The acquisition at each bias field is then processed with the reference measurement; both measurements are smoothed using a Gaussian weighted filter, to remove most of the high frequency noise. Then, any drift in the voltage of the waveforms between the reference and data is minimised, as is any trigger difference. After subtracting the reference measurement from the data, the processional response of the magnetisation around the direction of the bias field H is obtained. By Fourier transform of the induced signal, we obtain the resonance frequency f, at each bias field. With this technique, we characterised both the BN sample and the Ni_80_Fe_20_ AD reference sample, but we have been unable to measure the Co dot sample, since in this setup it does not generate a large enough signal.

Micromagnetic simulations have been performed with the MuMax3.9 software (DyNaMat Group Ghent University, Ghent, Belgium) on a 2.6 μm × 2.6 μm grid repeated five times in each direction containing Co dots with a diameter of 350 nm and surrounded by a Ni_80_Fe_20_ matrix. Standard values for the properties of the magnetic materials have been used. Periodic boundary conditions have been imposed. The dots have been arranged in a disordered configuration closely resembling typical arrangements observed in SEM images of similar size.

## 3. Results and Discussion

**Figure 3:**
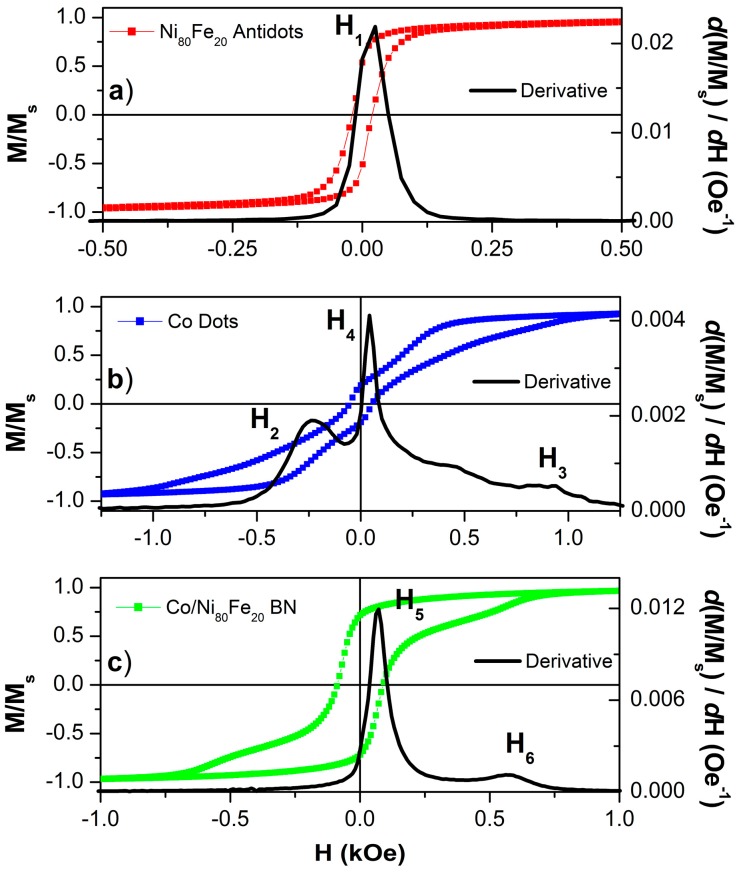
(Colour online) Room temperature, in-plane hysteresis loops of Ni_80_Fe_20_ antidot arrays (**a**); Co dot arrays (**b**) and bi-component nanostructured systems (**c**). Black lines are the field derivative of the lower branch of the M/M_S_ experimental values. Horizontal scales have been adjusted to put in evidence the features of the loops for the different samples.

The room temperature hysteresis loop of the reference Ni_80_Fe_20_ antidot array is reported in Figure 3a. A reduced remanence ratio of M_r_/M_s_ = 0.7 and a coercive field value of H_c_ = 19 Oe characterise this sample, whose hysteresis loop is not too different from that of a Ni_80_Fe_20_ continuous thin film with the same thickness (not shown here, [31]). However, the sample’s hystersis loop has a larger coercivity to be ascribed to the anisotropy energy contribution deriving from the holes array, which forces the magnetisation either to rotate around them, or to form free poles at their edges [31,34]. The field H_1_, at which the most significant magnetisation reversal process takes place, can be put in evidence by plotting the derivative of the magnetisation curve with respect to the applied field (solid line in Figure 3a). For convenience of comparison with FORC distributions, the derivative of the ascending (lower) branch has been calculated. In the Ni_80_Fe_20_ antidot array, H_1_ is located in correspondence of the coercive field.

In Figure 3b, the hysteresis loop of the reference Co dot array is shown: its shape is typical of those systems where a magnetic vortex nucleates at sufficiently low applied fields, and then moves as the field is progressively reversed until the vortex is expelled. This result is expected, as Co dots with comparable size and thickness have been reported to show such behaviour [35,36]. The magnetisation jumps that correspond to the vortex nucleation and annihilation are clearly visible, and are marked with the fields H_2_ and H_3_ in the field derivative of the lower loop branch. Contrary to what is often observed in dot arrays, a relatively large hysteresis is observed close to zero applied field (M_r_/M_s_ = 0. 19 and H_c_ = 55 Oe); in proximity of the coercive field, a significant magnetisation reversal process occurs, identified by H_4_. The presence of a non-vanishing coercive field is unexpected in non-interacting dot arrays displaying a vortex domain configuration. However, coercivity may be seen to increase in the presence of magnetic interactions among the dots caused by the partial disorder of the array, where dots may sometimes be very close or even touch (see the SEM image of Figure 1f for a representative example on the BN sample).

Finally, the magnetisation reversal of the two-dimensional bi-component nanostructured array Co/Ni_80_Fe_20_ is reported in Figure 3c. When the applied magnetic field is reduced from saturation to zero, the magnetisation remains almost constant (M_r_/M_s_ = 0.7), as in the Ni_80_Fe_20_ antidot reference sample. When the applied magnetic field is further reduced and reverses, a rapid magnetisation jump appears, not dissimilar to the magnetisation reversal of the antidot reference sample, even if the coercive field (H_c_ = 89 Oe) is significantly larger (peak identified as H_5_). Further increase of the magnetic field towards saturation in the opposite direction brings a reversal process that resembles that of the Co dots (vortex expulsion), with a minor peak H_6_ in the derivative of the magnetisation curve that is located at a field with lower absolute value with respect to H_3_. It is worth noting that the equivalent of the magnetisation jump occurring at H_2_ in the Co dot reference sample (vortex nucleation) is not observed in the BN sample. 

**Figure 4:**
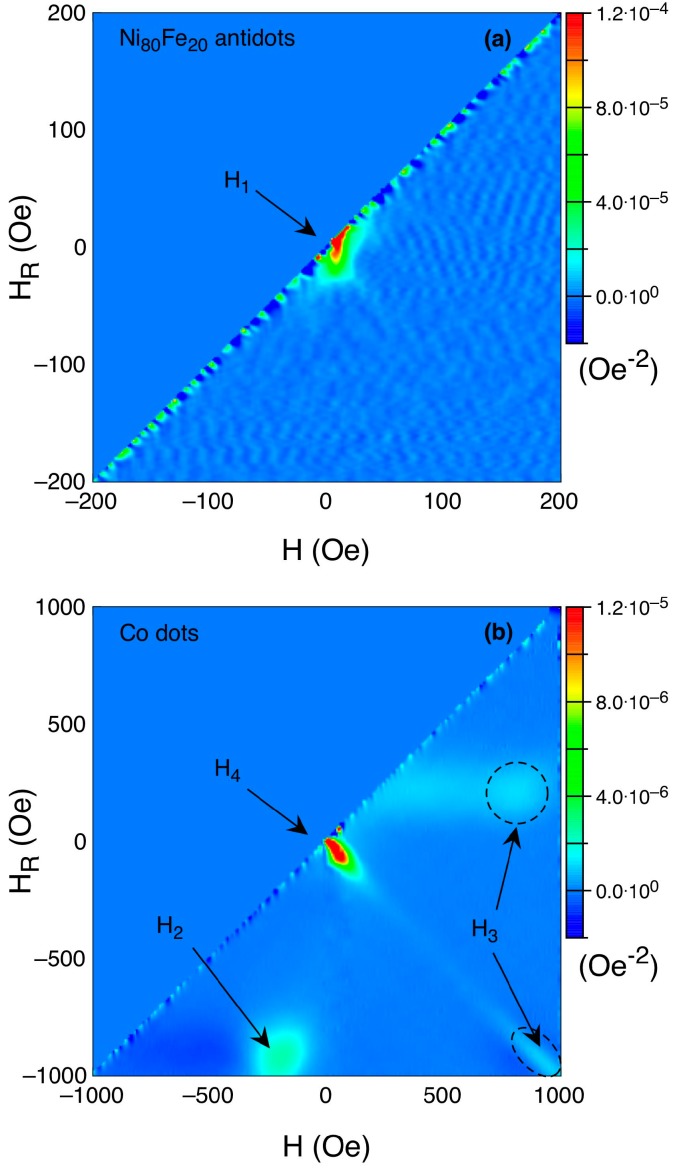

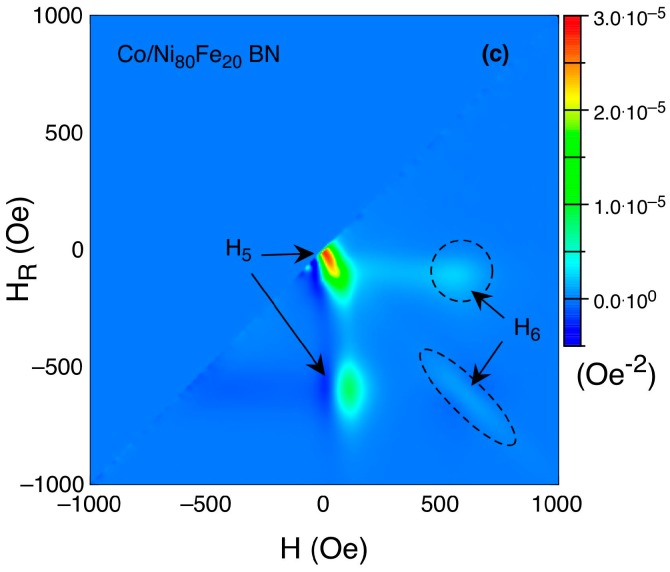
(Colour online) First order reversal curves (FORC) of Ni_80_Fe_20_ antidot arrays (**a**); Co dot arrays (**b**) and bi-component nanostructured systems (**c**). The colour plots represent the distribution of the second derivative of the normalised magnetisation with respect to the applied field, H, and the reversal field H_R_ (in Oe^−2^). Values of the distribution close to zero correspond to reversible magnetisation processes, whereas the values of the distributions different than zero correspond to irreversible magnetisation processes. Regions highlighted with dashed lines better put in evidence the features marked with H_3_ and H_6_.

The hysteresis loop of the BN sample is clearly not the linear combination of those of the two reference samples; therefore, the BN sample is not simply the superposition of the Ni_80_Fe_20_ AD and of the Co dot samples. This indicates that the two magnetic materials, the Co constituting the dots and the Ni_80_Fe_20_ constituting the antidot matrix, are not independent; rather, they are mutually interacting. To further investigate this aspect, we performed FORC measurements on all three samples, whose results are reported in Figure 4. FORCs are an effective means of identifying the irreversible processes (which give a signal) and distinguishing them from the reversible ones (which do not give any signal) occurring during the magnetisation reversal in magnetic samples [37,38,39].

The FORC distribution for the Ni_80_Fe_20_ reference sample (Figure 4a) is characterised by a not very sharp peak at very low applied field, indicating that the reversal of the magnetisation in this sample occurs with a sequence of irreversible jumps peaked at H_1_ (which is practically H_c_) and slightly distributed around this field value. This leads to the conclusion that the reversal process in the antidot array is mostly a large-scale process, where large regions of the sample reverse their magnetisation at the same time in single, irreversible jumps.

The FORC distribution of the Co dots (Figure 4b), conversely, is characterised by the presence of four irreversible features, clearly located. One is a sharp peak at low H and H_R_ values that corresponds to the H_4_ reversal process observed in Figure 3; this process could be ascribed to the reversal of the magnetisation in clusters of nearby interacting dots, which results from imperfections of the PN lithography process (as discussed earlier). Due to these imperfections, a collective reorientation of the magnetisation occurs without the nucleation of a magnetic vortex in the individual dots. Two other peaks coincide with the nucleation and expulsion of the vortex [40,41] (H_2_ and H_3_ respectively following the nomenclature of Figure 3). Of these, the sharpest (negative H) coincides with the nucleation, whereas the one located on the same horizontal line (positive H, negative H_R_) coincides with the expulsion at the opposite edge of the dot with respect to the nucleation. Finally, a weaker peak measured by the reversal curves at positive H_R_ values, therefore symmetrically located with respect to the nucleation one, marks the expulsion along the same dot edge where nucleation has occurred [41].

As already discussed for the hysteresis loops, the FORC diagram of the BN sample (Figure 4c) is not simply the superposition of those of the reference samples; rather, it has distinct features, even if it may resemble that of the Co dots. The peak located at small H and H_R_ values coincides with the large magnetisation jump H_5_ detected by the hysteresis loop. In the case of the Ni_80_Fe_20_ antidot sample, this peak corresponded to the large-scale magnetisation reversal in the antidot array in close proximity of the coercive field. In the case of the BN sample, however, at the same positive H values a sharp peak located at negative H_R_ values appears. In analogy with the case of the Co dots, this peak can be interpreted as the nucleation of the vortex in the Co dots in the BN sample. However, in this case, the nucleation is triggered by the reversal process in the antidot matrix, which indicates a significant coupling between the two magnetic components of the BN sample. Therefore, the vortex does not nucleate in the Co dots of the BN sample before reaching the magnetic remanence. As for the Co dots alone, at remanence the Co dots are still almost saturated. This is indicated by the large remanence value of the loop reported in Figure 3c, and only in the correspondence of the coercive field when the magnetisation in the Ni_80_Fe_20_ antidot matrix reverses, at which point the magnetisation in at least in a few of the dots develops a vortex structure. In the hysteresis loop, these two processes occur simultaneously and give rise to the single H_5_ peak, whereas the FORC distribution clearly detects them individually. When the field is further increased, then, the vortex expulsion peaks appear both in the loop and in the FORC distribution, but with significantly reduced H values. These values indicate again a strong magnetic coupling between the two magnetic components of the BN sample, with the antidot matrix forcing the dots to saturation earlier than in the case of the isolated dots.

**Figure 5:**
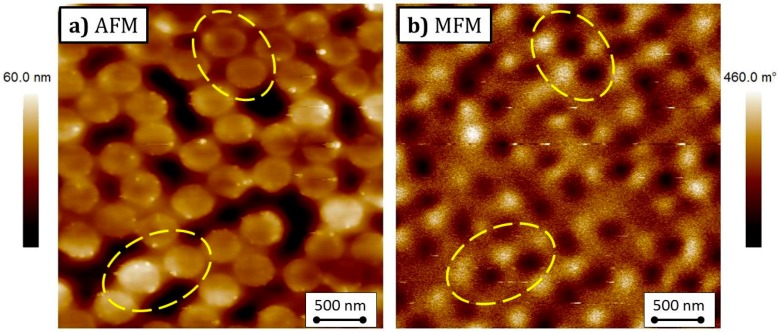
(Colour online) AFM image (**a**) and corresponding Magnetic force microscopy (MFM) image (**b**), taken at the magnetic remanence after saturation along the horizontal direction, of a portion of the bi-component nanostructured system. Highlighted areas put in evidence the Co dots whose magnetisation at zero applied field is close to the saturated state.

This picture is confirmed by the AFM/MFM images reported in Figure 5, where the magnetic component is taken at the remanence after having saturated the sample with an in-plane applied field. In the AFM image, the dots appear in a bright colour on a dark background. In fact, as discussed in Section 2, they are slightly thicker than the surrounding antidot matrix; this makes them clearly visible in the topographic image. The MFM image, as expected, does not show any features of vortices in the dots. Conversely, they are all magnetically saturated, as already discussed earlier: at zero applied field, vortex nucleation in the dots in the BN sample has yet to come, because of the interaction with the Ni_80_Fe_20_ antidot matrix. Two examples are put in evidence in the marked areas of Figure 5, with magnetic saturation indicated by the bright colour to the left, and the dark colour to the right.

**Figure 6:**
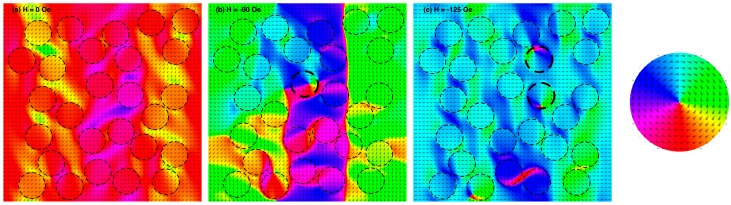
(Colour online) Micromagnetic simulations of a portion of a BN system with a size of 2.6 μm × 2.6 μm. Colours and arrows indicate the direction of the magnetisation (see legend to the right). The dashed circles identify the Co dots inside the Ni_80_Fe_20_ matrix. (**a**) At magnetic remanence after saturation at a positive field (aligned horizontally to the right); (**b**) at a field of −90 Oe (aligned horizontally to the left); (**c**) at a field of −125 Oe (aligned horizontally to the left). In (**b**,**c**), the thicker circles indicate dots where a vortex magnetisation has developed.

The above interpretation is further confirmed by the micromagnetic simulations; these results are summarised in Figure 6. The simulated portion of the BN sample has been saturated at positive field, then brought at remanence (Figure 6a). All the Co dots still appear saturated and strongly coupled with Ni_80_Fe_20_ matrix, which is in agreement with the MFM image of Figure 5. At a field of −90 Oe, i.e., very close to the measured coercive field (see Figure 3c), a significant reorientation of the magnetisation in the Ni_80_Fe_20_ antidot matrix takes place (Figure 6b). In the simulations, this event takes places in a few steps in a limited field range (Figure 6c), and is accompanied by a reorientation of the magnetisation of the Co dots. However, a few of them remain decoupled from the antidot matrix, and develop a vortex structure (dots identified with thicker borders in Figure 6), in agreement with the hysteresis loops and FORC and MFM measurements. It is interesting to point out that if the simulations are carried out for ordered arrays, a more homogeneous behaviour takes place, with a sharper inversion of the magnetisation with respect to the applied field and a stronger coupling of the Co dots with the Ni_80_Fe_20_ antidot matrix. The vortex nucleation in some dots occurring at the same time that the antidot matrix reverses its magnetisation seems therefore particularly enhanced by the presence of defects, dislocations in the lattice, and disorder in these systems prepared by nanospheres lithography.

The magnetic behaviour of the three samples has also been investigated at low temperature by means of hysteresis loops measurements performed in zero-field cooled (ZFC) condition. The samples have been demagnetised at room temperature by applying an oscillating magnetic field of progressively decreased amplitude, and then cooled in zero applied field to the minimum temperature. From there, hysteresis loops have been measured in sequence, with increasing temperature.

**Figure 7:**
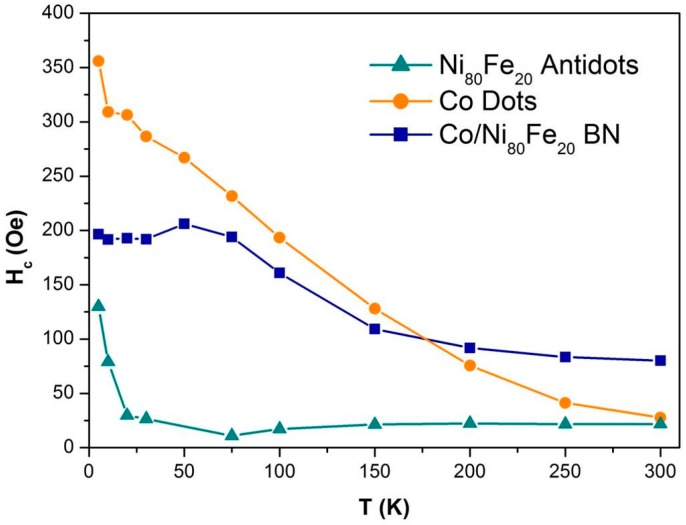
(Colour online) Temperature dependence of the coercive field of Ni_80_Fe_20_ antidot arrays, Co dot arrays, and bi-component nanostructured systems.

Figure 7 reports the coercivity values obtained from these measurements. In the antidot sample, the shape of the hysteresis loop remains practically the same in the whole temperature range, with just a small increase of coercivity below 25 K that does not seem to modify the magnetisation reversal mechanisms. However, on the dots sample, there is an observed steady increase of the coercive field across the whole temperature range upon reducing T. Indeed, this has to be ascribed to the H_4_ peak of Figure 3b. The vortex nucleation and expulsion fields remain almost unaltered by the temperature reduction, with just an expected slight increase on decreasing the sample temperature. The dot clusters, on the other hand, not showing vortex magnetisation and being responsible for the H_4_ peak become progressively magnetically harder as the temperature is reduced. This can be ascribed to the magnetic interaction among these dots, which becomes progressively stronger as the temperature decreases, as expected. In the case of the BN sample, at all temperatures the description given for Figure 3c still holds. The coercive field larger than those of the Co dots and the Ni_80_Fe_20_ antidot matrix has to be ascribed to the coupling between the two magnetic materials. The dots do not nucleate a vortex magnetisation until a sufficiently large magnetic field opposite to the initial saturation is applied, which forces the two magnetic components to decouple. At this point, the antidot matrix reverses its magnetisation (with a relatively large magnetic field), and the Co dots develop a vortex structure. On decreasing temperature, the picture remains practically the same, but the coercivity of the dot clusters progressively increases. However, in the BN sample also, these dot clusters are magnetically coupled with the antidot matrix, giving rise to a coercivity increase that is in-between the almost zero variation observed in the Ni_80_Fe_20_ antidots and the steady variation of the Co dots.

**Figure 8:**
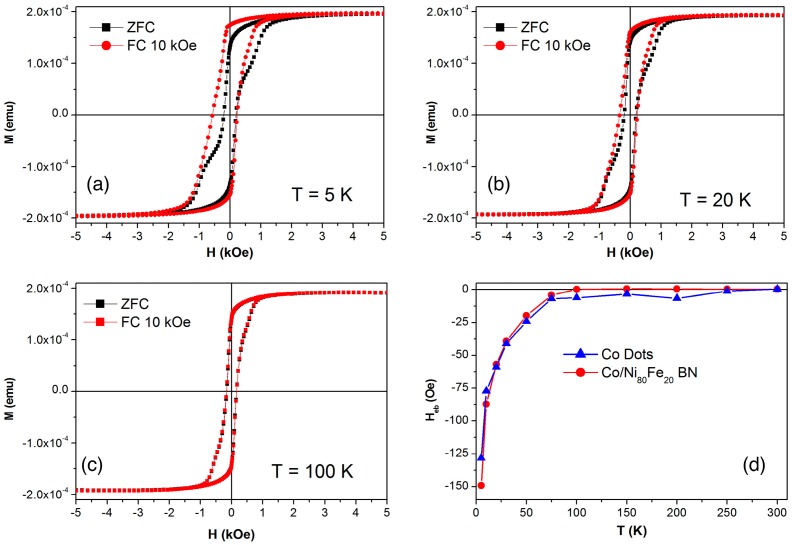
(Colour online) Hysteresis loops of the bi-component nanostructured system measured at 5, 20 and 100 K (panels (**a**)–(**c**) respectively), in field-cooled under 10 kOe applied field and zero-field cooled conditions. (**d**) Temperature dependence of the exchange bias field in Co dot arrays and bi-component nanostructured systems.

The picture becomes more complex when the hysteresis loops are measured as a function of temperature in a field cooled condition, i.e., with the sample brought to the minimum temperature from room temperature under the application of a saturating magnetic field of +10 kOe. Examples of hysteresis loops in the BN sample are reported in Figure 8 for three selected temperatures. It can be observed that as the temperature is reduced, the loops measured in the ZFC and FC conditions become progressively different, and the latter is also significantly asymmetric. This effect is common in exchange-biased systems, where a ferromagnetic and antiferromagnetic layer are exchange coupled through their interface. In such systems, the cooling in the magnetic field below the Néel temperature of the antiferromagnet causes its alignment to the cooling field, while at the interface with the ferromagnet a preferential orientation of its magnetisation is imposed by the exchange coupling between the two materials. As a result, the hysteresis loops become deformed and asymmetric below the Néel temperature, as the interaction field at the interface is superimposed to the applied field [15,16].

In our case, an intrinsic antiferromagnetic layer develops spontaneously in the Co dots, both alone and embedded in the Ni_80_Fe_20_ matrix. In fact, a native oxide layer develops on the top surface of the Co dots. Since Co-oxide is an antiferromagnet [42], it can be the source of this exchange bias effect [43]. Indeed, the bias field is reported in Figure 8d for the Co dots and the BN samples, showing that the effect is identical. No bias field is observed for the Ni_80_Fe_20_ antidot sample. The bias field changes sign when the cooling is done under a field with the opposite sign (not shown here), while the Néel temperature below 75 K is compatible with a nanostructured Co-oxide [44,45] that probably has a thickness of just a few nanometers, and a sub-optimal interface coupling between Co and Co-oxide, since the latter develops spontaneously through natural oxidation in the air.

**Figure 9:**
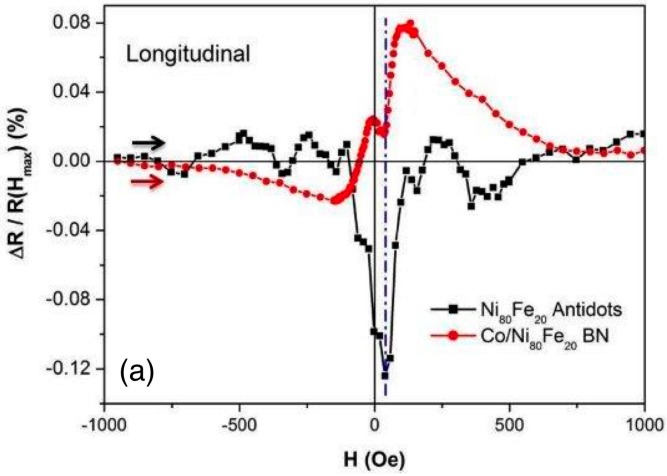

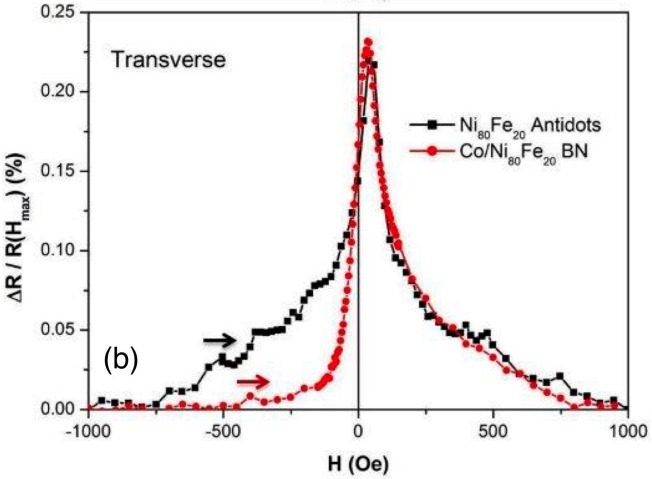
(Colour online) Room temperature magnetoresistance of Ni_80_Fe_20_ antidot arrays and of bi-component nanostructured systems in longitudinal (**a**) and transverse (**b**) configuration. Arrows indicate the field span direction.

Magneto-resistance measurements have also been performed on the Ni_80_Fe_20_ antidot sample and the BN sample, both in the longitudinal (applied field parallel to electrical current) and transverse (applied field perpendicular to electrical current, but still in the sample plane) configurations. Data on Co dots are missing because they do not constitute a percolating array; therefore, magneto-resistance measurements cannot be performed. Figure 9 reports MR data at room temperature as a function of H: the field starts at negative values and progressively increases to zero, and then to positive values. For the Ni_80_Fe_20_ antidot sample, the measured curves represent an anisotropic magneto-resistance (AMR) effect [31], which is typical in these systems. In the case of the longitudinal configuration, at high negative field values the magnetisation is saturated in the field direction, which gives rise to a certain resistance value. As the field approaches zero, the magnetisation progressively rotates around the holes in the antidot structure to minimise the magnetostatic energy. As a result, the magnetisation becomes progressively more parallel to the current density, and an increase of the resistance value should result. However, as Figure 3a shows, the magnetisation of the antidot array starts decreasing toward zero applied field, indicating that domains with different orientations appear. The domains will have their magnetisation statistically aligned in a different direction than the applied current, and an overall effect of decreased resistance is observed. On inverting the field sign and reaching coercivity, a magnetic configuration of maximum misalignment of the magnetisation with respect to the current is obtained. This results in a minimum of the resistance that eventually restores its saturation value when the applied field increases. A similar explanation holds for the transverse configuration: when a large negative field is applied, the magnetisation is mostly perpendicular to the current density, resulting in a low resistance value. When the field is reduced toward zero, the magnetisation progressively rotates away from the initial direction as more domains are formed, and an overall increase of the resistance results. Therefore, an upwards peak is obtained at the coercivity, where the maximum domain fragmentation results in an increased probability of having the magnetisation locally aligned to the current.

In the case of the BN sample, the main effect is still to be ascribed to AMR, but the magnetisation configuration is modified by the presence of the two coupled magnetic materials. The transverse configuration is mostly the same as for the Ni_80_Fe_20_ antidot array, with the notable exception that the MR curve is flatter for the BN material until the field is reduced much closer to zero. In fact, the magnetisation remains relatively aligned to the applied transverse field at the same time thanks to the coupling between the dots and the surrounding antidot matrix, and the current is not significantly forced into curved paths as it can also flow in the Co dots (which in the antidot samples were instead holes). Therefore, the orthogonal configuration of current density and magnetisation holds for a larger field interval than for the AD sample, until the fragmentation into domains causes the appearance of the positive peak and the progressive recovery to saturation with a MR evolution equivalent to that of the antidot array. In the longitudinal case, instead, a more complex field evolution of the electrical resistance takes place. A behaviour similar to the antidot sample should at first be expected, in analogy to the transverse configuration. Conversely, even though a local minimum is detected in the correspondence of the coercive field located at H_5_ (see Figure 3c and dashed line in Figure 9), a significant increase of the resistance around this minimum is also detected. In the framework of the anisotropic magnetoresistance, an increase of the resistance value is associated to a more ordered magnetic configuration that is parallel to the direction of the applied field. However, as this same configuration is supposedly obtained at saturation in longitudinal configuration (in Figure 9, the maximum applied is 1000 Oe), the reported increase of the resistance during the magnetisation reversal in BN systems in the longitudinal configuration has to be ascribed to different mechanisms. Indeed, magnetic scattering of the conduction electrons at the interface between the antidot array and the Co dots can also be envisaged, with a giant magnetoresistance-like (GMR) mechanism. Within this framework, a low scattering probability (i.e., a low contribution to the total resistance) is expected when the magnetisation of the antidot matrix and the Co dots is parallel. Upon reduction of the field toward zero, the magnetisation in the dots will begin to slightly misalign from that of the antidot matrix due to the tendency of the dots to form magnetic vortices. When the two magnetisations decouple, with that of the antidot matrix reversing and that of the Co dots forming vortices (see Figure 3b), a significant degree of misalignment between them will be present, which contributes with a scattering probability that results in an increased resistance value. In our system, the size of the Co dots and their average distance are quite large compared to optimised GMR systems (where the size of the magnetic features, e.g., layers, should be comparable with the conduction electron mean free path), which results in a weakly efficient GMR-like effect. In fact, this effect is barely able to compensate for the AMR contribution and result in a positive resistance variation having an amplitude of less than 0.1%, in agreement with the minority nature of the magnetic scattering at the interface between the antidot matrix and the Co dots. Interestingly, the same effect should be observed in the transverse configuration; however, it adds to the AMR contribution (both are positive in this case), therefore resulting in a single, positive peak.

**Figure 10:**
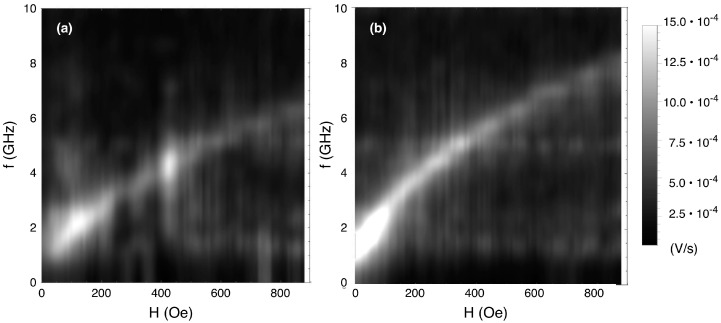
Dispersion measurement in the Ni_80_Fe_20_ antidot array (**a**) and in the bi-component nanostructured system (**b**).

The interplay between the antidot matrix and the Co dots in the BN sample has also been studied by means of dynamic investigations. High frequency measurements of both the antidot (AD) sample and the bi-component (BN) sample show a single mode (Figure 10), with the mode in the antidot sample positioned at a lower frequency.

In ordered lattices with orthogonal symmetry, at least two modes have been observed in BN (AD) systems [46]: a lower frequency mode corresponding to a localised (extended) mode, and a higher frequency mode corresponding to an extended (localised) mode. This inversion of the modes between the two systems is explained by taking into account the sign of the demagnetising field in the regions between the dots, and in the columns between the columns of dots. Moving from the BN system to the AD system, both regions change sign. In the antidot sample, the holes are effectively constituted by a non-magnetic material, whereas in the BN sample, the holes are filled by another magnetic material that inhibits the formation of free poles (and therefore of demagnetising fields) among the holes. Some weak vibrational (at ~400 Oe) and electromagnetic (at ~1.6 and ~5 GHz) noise are visible, which do not affect the discussion of the measurement.

Our system differs both in being based on a hexagonal (and not orthogonal) geometry, and on having a disordered structure. Yet it was shown in [47] that the modes developed in hexagonal geometry systems exhibit a good one-to-one correspondence to those present in orthogonal geometry systems. In our case, just a single mode is visible: by comparing with [46] it comes out that the mode in the AD system is at a lower frequency when compared with the BN system. This indicates that we are in the presence in both cases of the extended mode, while the localised mode is too faint to be detected. Indeed, on increasing frequency, the modes appear in the following order: BN localised, AD extended, BN extended, AD localised. This is consistent with [47], where a similar set-up detected the extended mode in an antidot system, but not the localised one. The higher frequency of the BN mode with respect to the AD one is due to the strong contribution of the magnetisation of the Co dots, which increases the magnitude of the demagnetising field.

**Figure 11:**
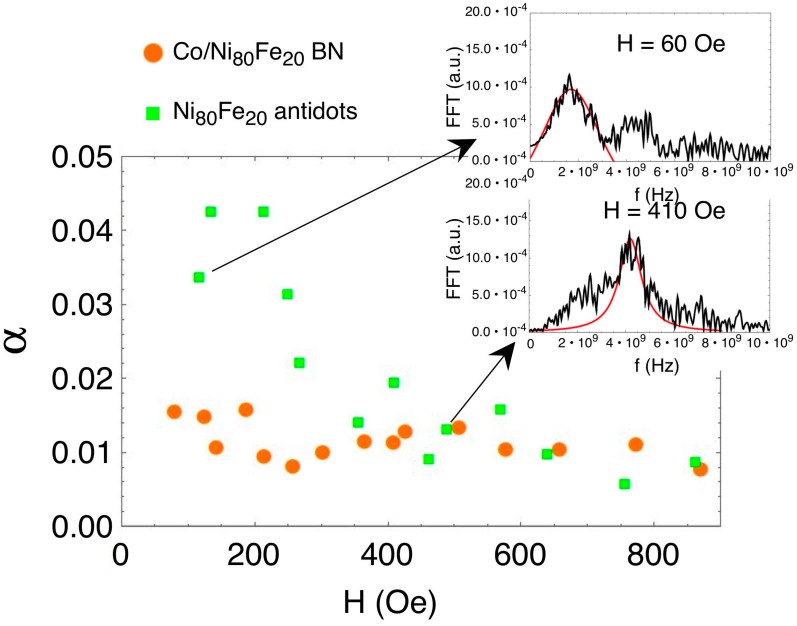
(Colour online) Damping constant α from measurement in a bi-component nanostructured system (orange) and Ni_80_Fe_20_ antidot array (green). Insets show examples of Lorentzian fits of the main mode of the antidot sample at 60 and 410 Oe, respectively.

The damping parameter α can then be obtained by the Lorentzian fitting of the main mode: the width Δ*f* is related to *α* by $\alpha =\frac{\pi ∆f}{\gamma \mu_{0}M_{S}}$ [46,48]. This measurement returns a value α ~ 0.01 in the whole field range 60 Oe < H < 900 Oe for the BN sample, whereas in the antidot sample the value increases up to α ~ 0.04 for H < 400 Oe (Figure 11).

The higher α value at low fields in the AD sample is possibly related to a higher noise level in the measurement in that field region. The value Δ*f* ~ 0.5 GHz for the AD sample is compatible with [46]; however, in their cases, the BN sample exhibits a higher damping with Δ*f* ~ 1.5 GHz, whereas in our case, the BN sample damping stays at the same value as the AD sample.

## 4. Conclusions

Bi-component nanostructured systems consisting of an array of Co dots dispersed in a Ni_80_Fe_20_ antidot matrix, and prepared by polystyrene nanospheres lithography, have been studied together with the respective systems consisting of either the dots or antidots elements. The magnetic properties of the bi-component nanostructured system turned out to be more complex than the superposition of the properties of its constituents. In particular, a strong interplay between the Ni_80_Fe_20_ matrix and the Co dots forced them to keep a saturated configuration at the magnetic remanence, whereas the Co dots alone would have developed a vortex magnetisation configuration. The reversal of the magnetisation in the Ni_80_Fe_20_ array, which is slightly delayed by the interaction with the Co dots, decouples the two subsystems of the bi-component sample. The dots acquire a magnetic vortex that is expelled at lower fields with respect to the Co dots alone, again due to the magnetic coupling with the magnetic matrix in which they are dispersed. The coupling between these two components is also responsible of the magnonic behaviour of this system, whose localised and extended modes are reversed with respect to typical antidot samples because of the added contribution of the Co dots to the demagnetising field.
